# Supplementary material for: Restriction of essential amino acids dictates the systemic metabolic response to dietary protein dilution
Source: Nat Commun. 2020 Jun 9;11:2894. doi: 10.1038/s41467-020-16568-z (PMC7283339; doi:10.1038/s41467-020-16568-z)
Supplement: Supplementary file 1 — Supplementary Information [file 41467_2020_16568_MOESM1_ESM.pdf]

Supplementary Table 1. Diet formulations from Specialty Feeds.

Ingredient	Diet Number<br>SF17-180<br>g/Kg	SF17-175	SF17-176	SF17-177	SF17-179	SF18-066	SF18-109	SF18-110	SF14-162	SF17-144	SF17-115	SF19-086
Sucrose	333.14	237.92	337.78	340.17	339.93	340.11	340.17	340.17	100	100	100	340.12
Casein	190.37	47.58	48.25						200	34.88	159.5	
L Methionine	2.86	0.71	3.65	4.9	5.05	4.9	1.22	1.03	3	0.78	12.48	1.22
L Alanine			3.65	4.9	5.05	4.9	1.22	1.03		3.47		7.52
L Arginine			4.28	5.74	5.92	5.74	1.44	1.22		4.51		8.82
L Asparagine			5.03	6.75	6.96	6.75	1.69	1.43		3.74		10.38
L Aspartic Acid			3.65	4.9	5.05	4.9	1.22	1.03		4		7.52
L Cystine			1.61	1.19	1.22	1.19	0.3	0.25		0.24		1.82
L Glutamic Acid			14.97	20.1	20.73	20.1	5.02	4.26		13.4		30.86
L Glutamine			12.32	16.55	17.07	16.55	4.14	3.51		11.27		25.42
Glycine			2.13	2.87	2.95	2.87	0.72	0.61		2.22		4.41
L Histidine			3.27	4.39	4.53	4.39	1.1	0.93		3.22		1.1
L Isoleucine			5.4	7.26	7.49	7.26	1.82	1.54		7.83	1.89	1.82
L Leucine			11.32	15.2	15.68	15.2	3.8	3.22		14.26	3.48	3.8
L Lysine			9.44	12.67	13.07	12.67	3.17	2.69		8.96		3.17
L Phenylalanine			6.04	8.11	8.36	8.1	2.03	1.72		5.83		2.03
L Proline			12.7	17.06	17.59	17.05	4.27	3.62		12.31		26.2
L Serine			7.17	9.63	9.94	9.63	2.41	2.04		6.62		14.79
L Threonine			5.15	6.92	1.73	1.73	1.73	6.29		0.23	9.61	1.73
L Tryptophan			1.51	2.02	2.09	2.02	0.51	2.02			2.03	0.51
L Tyrosine			6.54	8.79	9.06	8.78	2.2	1.87		6.24		13.49
L Valine			6.66	8.94	9.94	8.94	2.24	1.9		10.44	2.54	2.24
L Homoserine						5.35						
Soybean Oil	23.8	23.79	24.13	24.3	24.28	24.29	24.29	24.29	70	70	70	24.29
Lard	19.04	19.03	19.3	19.44	19.42	19.43	19.44	19.44				19.44
Cellulose	47.59	47.58	48.25	48.59	48.56	48.59	48.59	48.59	50	90.65	63.24	48.59
Wheat Starch	299.83	501.78	304	306.15	305.95	306.11	423.07	423.15	404.41	403.19	403.91	306.11
Dextrinised Starch	33.31	71.38	33.78	34.02	33.99	34.01	43.74	43.73	132	132	132	34.01
AIN93 Trace Minerals	1.33	1.33	1.35	1.36	1.36	1.36	1.36	1.36	1.4	1.4	1.4	1.36
Calcium Carbonate	5.24	5.23	5.31	5.35	5.34	5.34	5.35	5.34	13.13	13.1	13.13	5.34
Salt	2.47	2.47	2.51	2.53	2.53	2.53	2.53	2.53	2.59	2.59	2.59	2.53
Potassium Dihydrogen Phosphate									6.86	10.11	6.04	
Sodium Bicrbonate			7.24	7.29	7.28	7.29	7.29	7.29				7.29
Potassium Sulphate	1.52	1.52	1.54	1.56	1.55	1.55	1.56	1.56	1.63	7.53	1.18	1.55
Potassium Citrate	15.71	15.71	15.92	16.04	16.03	16.03	16.04	16.03	2.48	2.48	2.48	16.03
Dicalcium Phosphate	12.37	12.37	12.55	12.63	12.63	12.63	12.63	12.63				12.63
AIN 93 Vitamins	9.52	9.52	9.65	9.72	9.71	9.72	9.72	9.72	10	10	10	9.72
Choline Chloride 75%	1.9	1.91	1.93	1.94	1.94	1.94	1.94	1.94	2.5	2.5	2.5	1.94
Red Food Colour (124)		0.02	0.02									
Blue Food Colour 10% (133)		0.15										0.19
Egg Yellow Food Colour (102)				0.02								
Green Food Colour (133, 102)					0.02			0.02				
Sunset Yellow Food Colour (110)						0.05	0.03					
Total	1000	1000	1000	1000	1000	1000	1000	1000	1000	1000	1000	1000

Supplementary Table 2. Diet formulations from Research Diets.

Product #	A14011801		A14011802		A14011803		A14011804		A14011806		A14011808	
	100% AA		25% AA		25% EAA, 100% NE		100% EAA, 25% NE		25% EAA, 154% NE		205% EAA, 25% NE	
	41.7% EAA, 58.3% NE		41.7% EAA, 58.3% NE		15.2% EAA, 84.8% NE		74.1% EAA, 25.9% NE		10.4% EAA, 89.6% NE		85.4% EAA, 14.6% NE	
	gm	kcal	gm	kcal	gm	kcal	gm	kcal	gm	kcal	gm	kcal
Protein	16.8	17.6	4.2	4.4	11.6	12.1	9.5	9.9	16.8	17.6	16.8	17.6
Carbohydrate	68.7	72.1	81.3	86.3	74.0	77.6	76.1	79.6	68.7	72.1	68.7	72.1
Fat	4.4	10.3	4.4	10.3	4.4	10.3	4.4	10.3	4.4	10.3	4.4	10.3
Total		100.0		100.0		100.0		100.0		100.0		100.0
kcal/gm	3.8		3.8		3.8		3.8		3.8		3.8	
Ingredient (gm)												
L-Histidine-HCl-H2O	4.52	18.08	1.13	4.5	1.13	4.5	4.52	18.08	1.13	4.5	9.25	37.0
L-Isoleucine	7.47	29.88	1.87	7.5	1.87	7.5	7.47	29.88	1.87	7.5	15.31	61.2
L-Leucine	15.64	62.56	3.91	15.6	3.91	15.6	15.64	62.56	3.91	15.6	32.03	128.1
L-Lysine-HCl	13.04	52.16	3.26	13.0	3.26	13.0	13.04	52.16	3.26	13.0	26.70	106.8
L-Methionine	5.04	20.16	1.26	5.0	1.26	5.0	5.04	20.16	1.26	5.0	10.32	41.3
L-Phenylalanine	8.34	33.36	2.09	8.4	2.09	8.4	8.34	33.36	2.09	8.4	17.08	68.3
L-Threonine	7.12	28.48	1.78	7.1	1.78	7.1	7.12	28.48	1.78	7.1	14.59	58.4
L-Tryptophan	2.08	8.32	0.62	2.1	0.62	2.1	2.08	8.32	0.62	2.1	4.27	17.1
L-Valine	9.20	36.8	2.30	9.2	2.30	9.2	9.20	36.8	2.30	9.2	18.86	75.4
L-Alanine	5.04	20.16	1.26	5.0	5.04	20.2	1.26	5.04	7.74	31.0	1.26	5.0
L-Arginine	5.91	23.64	1.48	5.9	5.91	23.6	1.48	5.92	9.08	36.3	1.48	5.9
L-Asparagine-H2O	6.95	27.8	1.74	7.0	6.95	27.8	1.74	6.96	10.68	42.7	1.74	7.0
L-Aspartate	5.04	20.16	1.26	5.0	5.04	20.2	1.26	5.04	7.74	31.0	1.26	5.0
L-Cystine	1.22	4.88	0.31	1.2	1.22	4.9	0.31	1.24	1.87	7.5	0.31	1.2
L-Glutamine	17.03	68.12	4.26	17.0	17.03	68.1	4.26	17.04	26.16	104.6	4.26	17.0
L-Glutamic Acid	20.68	82.72	5.17	20.7	20.68	82.7	5.17	20.68	31.76	127.0	5.17	20.7
Glycine	2.95	11.8	0.74	3.0	2.95	11.8	0.74	2.96	4.54	18.2	0.74	3.0
L-Proline	17.55	70.2	4.39	17.6	17.55	70.2	4.39	17.56	26.96	107.8	4.39	17.6
L-Serine	9.91	39.64	2.48	9.9	9.91	39.6	2.48	9.92	15.22	60.9	2.48	9.9
L-Tyrosine	9.04	36.16	2.26	9.0	9.04	36.2	2.26	9.04	13.88	55.5	2.26	9.0
Corn Starch	315	1260	435.2	1741	359.2	1437	381	1524	315	1260	315	1260
Maltodextrin 10	35	140	45	180	45	180	45	180	35	140	35	140
Sucrose	350	1400	350	1400	350	1400	350	1400	350	1400	350	1400
Cellulose	50	0	50	0	50	0	50	0	50	0	50	0
Soybean Oil	25	225	25	225	25	225	25	225	25	225	25	225
Lard	20	180	20	180	20	180	20	180	20	180	20	180
Sodium Bicarbonate	7.5	0	7.5	0	7.5	0	7.5	0	7.5	0	7.5	0
S10026	10	0	10	0	10	0	10	0	10	0	10	0
DiCalcium Phosphate	13	0	13	0	13	0	13	0	13	0	13	0
Calcium Carbonate	5.5	0	5.5	0	5.5	0	5.5	0	5.5	0	5.5	0
Potassium Citrate, 1 H2O	16.5	0	16.5	0	16.5	0	16.5	0	16.5	0	16.5	0
Vitamin Mix V10001	10	40	10	40	10	40	10	40	10	40	10	40
Choline Bitartrate	2	0	2	0	2	0	2	0	2	0	2	0
FD&C Yellow Dye #5	0.05	0	0.025	0	0	0	0.025	0	0	0	0	0
FD&C Red Dye #40	0	0	0.025	0	0.05	0	0	0	0	0	0.025	0
FD&C Blue Dye #1	0	0	0	0	0	0	0.025	0	0.05	0	0.025	0
Total	1033.32	3940	1033.22	3940	1033.19	3940	######	3940	1033.30	3940	1033.31	3940
Percent EAA (w/w)	41.7		41.7		15.2		74.1		10.4		85.4	
Percent NEAA (w/w)	58.3		58.3		84.8		25.9		89.6		14.6	

Product #	A18120601		A18120602		A18120503	
	19.3% EAA, 80.7% NE		17.5% EAA, 82.5% NE		23.5% EAA, 76.5% NE	
	gm	kcal	gm	kcal	gm	kcal
Protein	16.8	17.6	16.8	17.6	16.8	17.6
Carbohydrate	68.7	72.1	68.7	72.1	68.7	72.1
Fat	4.4	10.3	4.4	10.3	4.4	10.3
Total		100.0		100.0		100.0
kcal/gm	3.8		3.8		3.8	
Ingredient (gm)						
L-Histidine-HCl-H2O	1.02	4.1	4.62	18.1	0.96	3.8
L-Isoleucine	1.68	6.7	1.72	6.9	7.47	29.9
L-Leucine	3.52	14.1	3.60	14.4	15.64	62.6
L-Lysine-HCl	13.04	52.2	3.00	12.0	2.78	11.1
L-Methionine	1.14	4.6	5.04	20.2	1.08	4.3
L-Phenylalanine	1.88	7.5	8.34	33.4	1.78	7.1
L-Threonine	7.12	28.5	1.64	6.6	1.52	6.1
L-Tryptophan	2.08	8.3	0.48	1.9	0.44	1.8
L-Valine	2.07	8.3	2.12	8.5	9.20	36.8
L-Alanine	6.97	27.9	7.13	28.5	6.61	26.4
L-Arginine	8.18	32.7	8.36	33.4	7.75	31.0
L-Asparagine-H2O	9.62	38.5	9.83	39.3	9.12	36.5
L-Aspartate	6.97	27.9	7.13	28.5	6.61	26.4
L-Cystine	1.68	6.7	1.72	6.9	1.60	6.4
L-Glutamine	23.57	94.3	24.09	96.4	22.33	89.3
L-Glutamic Acid	28.61	114.4	29.24	117.0	27.11	108.4
Glycine	4.09	16.4	4.18	16.7	3.88	15.5
L-Proline	24.29	97.2	24.82	99.3	23.02	92.1
L-Serine	13.71	54.8	14.01	56.0	12.99	52.0
L-Tyrosine	12.50	50.0	12.78	51.1	11.85	47.4
Corn Starch	315	1260	315	1260	315	1260
Maltodextrin 10	35	140	35	140	35	140
Sucrose	350	1400	350	1400	350	1400
Cellulose	50	0	50	0	50	0
Soybean Oil	25	225	25	225	25	225
Lard	20	180	20	180	20	180
Sodium Bicarbonate	7.5	0	7.5	0	7.5	0
S10026	10	0	10	0	10	0
DiCalcium Phosphate	13	0	13	0	13	0
Calcium Carbonate	5.5	0	5.5	0	5.5	0
Potassium Citrate, 1 H2O	16.5	0	16.5	0	16.5	0
Vitamin Mix V10001	10	40	10	40	10	40
Choline Bitartrate	2	0	2	0	2	0
FD&C Yellow Dye #5	0	0	0.025	0	0	0
FD&C Red Dye #40	0.05	0	0	0	0.025	0
FD&C Blue Dye #1	0	0	0.025	0	0.025	0
Total	1033.29	3940	1033.3	3940	1033.29	3940
Percent EAA (w/w)	19.3		17.5		23.5	
Percent NEAA (w/w)	80.7		82.5		76.5	

Product #	A17020801		A17020802		A17020803		A17041301		A17041302		A17041303	
	100% Lys		100% Thr		100% Trp		Low Lys		Low Thr		Low Trp	
	15.5% EAA, 84.5% NE		13.2% EAA, 86.8% NE		11.2% EAA, 88.8% NE		38.1% EAA, 61.9% NE		39.8% EAA, 60.2% NE		41.2% EAA, 58.8% NE	
	gm	kcal	gm	kcal	gm	kcal	gm	kcal	gm	kcal	gm	kcal
Protein	16.9	17.7	16.8	17.7	16.8	17.6	16.8	17.6	16.8	17.6	16.8	17.6
Carbohydrate	68.7	72.0	68.7	72.1	68.7	72.1	68.7	72.1	68.7	72.1	68.7	72.1
Fat	4.4	10.3	4.4	10.3	4.4	10.3	4.4	10.3	4.4	10.3	4.4	10.3
Total		100.0		100.0		100.0		100.0		100.0		100.0
kcal/gm	3.8		3.8		3.8		3.8		3.8		3.8	
Ingredient (gm)												
L-Histidine-HCl-H2O	1.07	4.3	1.10	4.4	1.12	4.5	4.80	19.2	4.66	18.6	4.56	18.2
L-Isoleucine	1.77	7.1	1.81	7.2	1.85	7.4	7.92	31.7	7.71	30.8	7.54	30.2
L-Leucine	3.70	14.8	3.79	15.2	3.88	15.5	16.59	66.4	16.14	64.6	15.78	63.1
L-Lysine-HCl	13.04	52.2	3.16	12.6	3.23	12.9	3.26	13.0	13.46	53.8	13.16	52.6
L-Methionine	1.19	4.8	1.22	4.9	1.25	5.0	5.35	21.4	5.20	20.8	5.09	20.4
L-Phenylalanine	1.98	7.9	2.03	8.1	2.07	8.3	8.85	35.4	8.61	34.4	8.42	33.7
L-Threonine	1.69	6.8	7.12	28.5	1.76	7.0	7.55	30.2	1.78	7.1	7.18	28.7
L-Tryptophan	0.49	2.0	0.50	2.0	2.08	8.3	2.21	8.8	2.15	8.6	0.62	2.1
L-Valine	2.18	8.7	2.23	8.9	2.28	9.1	9.76	39.0	9.49	38.0	9.28	37.1
L-Alanine	7.33	29.3	7.51	30.0	7.67	30.7	5.35	21.4	5.20	20.8	5.09	20.4
L-Arginine	8.60	34.4	8.81	35.2	9.00	36.0	6.27	25.1	6.10	24.4	5.96	23.8
L-Asparagine-H2O	10.11	40.4	10.36	41.4	10.58	42.3	7.37	29.5	7.17	28.7	7.01	28.0
L-Aspartate	7.33	29.3	7.51	30.0	7.67	30.7	5.35	21.4	5.20	20.8	5.09	20.4
L-Cystine	1.77	7.1	1.81	7.2	1.85	7.4	1.29	5.2	1.26	5.0	1.23	4.9
L-Glutamine	24.77	99.1	25.38	101.5	25.93	103.7	18.07	72.3	17.58	70.3	17.18	68.7
L-Glutamic Acid	30.07	120.3	30.81	123.2	31.48	125.9	21.94	87.8	21.34	85.4	20.87	83.5
Glycine	4.30	17.2	4.40	17.6	4.50	18.0	3.13	12.5	3.04	12.2	2.98	11.9
L-Proline	25.52	102.1	26.16	104.6	26.72	106.9	18.62	74.5	18.11	72.4	17.71	70.8
L-Serine	14.41	57.6	14.77	59.1	15.08	60.3	10.51	42.0	10.23	40.9	10.00	40.0
L-Tyrosine	13.14	52.6	13.47	53.9	13.76	55.0	9.59	38.4	9.33	37.3	9.12	36.5
Corn Starch	315	1260	315	1260	315	1260	315	1260	315	1260	315	1260
Maltodextrin 10	35	140	35	140	35	140	35	140	35	140	35	140
Sucrose	350	1400	350	1400	350	1400	350	1400	350	1400	350	1400
Cellulose	50	0	50	0	50	0	50	0	50	0	50	0
Soybean Oil	25	225	25	225	25	225	25	225	25	225	25	225
Lard	20	180	20	180	20	180	20	180	20	180	20	180
Sodium Bicarbonate	7.5	0	7.5	0	7.5	0	7.5	0	7.5	0	7.5	0
S10026	10	0	10	0	10	0	10	0	10	0	10	0
DiCalcium Phosphate	13	0	13	0	13	0	13	0	13	0	13	0
Calcium Carbonate	5.5	0	5.5	0	5.5	0	5.5	0	5.5	0	5.5	0
Potassium Citrate, 1 H2O	16.5	0	16.5	0	16.5	0	16.5	0	16.5	0	16.5	0
Vitamin Mix V10001	10	40	10	40	10	40	10	40	10	40	10	40
Choline Bitartrate	2	0	2	0	2	0	2	0	2	0	2	0
FD&C Yellow Dye #5	0.025	0	0.025	0	0	0	0.025	0	0.025	0	0	0
FD&C Red Dye #40	0.025	0	0	0	0.025	0	0.025	0	0	0	0.025	0
FD&C Blue Dye #1	0	0	0.025	0	0.025	0	0	0	0.025	0	0.025	0
Total	1034.01	3943	1033.60	3941	1033.31	3940	1033.33	3940	1033.31	3940	1033.32	3940
Percent EAA (w/w)	15.5		13.2		11.2		38.1		39.8		41.2	
Percent NEAA (w/w)	84.5		86.8		88.8		61.9		60.2		58.8	

Supplementary Table 3. Liver proteomic analysis of the presence of yeast THR1 and THR4 transcripts of mice on a normal control amino acid contain diet pre-treated with adeno-associated viruses to express yeast THR biosynthetic transcripts (AAV-yTHR1+4) or a negative control (AAV-GFP) in hepatocytes.

Protein name	No. of identified unique peptides from sample	
	Control	THR1/4 over-expressed
THR1	0	14
THR4	0	15

Supplementary Table 4. Liver amino acid concentrations of mice on a normal control amino acid contain diet and a diet low in Threonine (Low THR) pre-treated with adeno-associated viruses to express yeast THR biosynthetic transcripts (AAV-yTHR1+4) or a negative control (AAV-GFP) in hepatocytes. Data are nmol/g wet weight liver and are mean ± S.E.M; n=6 per group. Two-way ANOVA: effect of diet, *p<0.05; effect of AAV, #p<0.05. ME: main effect.

Amino acid	Normal Amino Acid		Low THR		ME
	AAV-GFP	AAV-yTHR1+4	AAV-GFP	AAV-yTHR1+4	
**Ala**	1730 ± 215	2507 ± 203	1582 ± 197	1910 ± 234	#
**Arg**	4.58 ± 0.45	5.58 ± 0.93	4.18 ± 0.33	4.84 ± 0.56	
**Asp**	212 ± 24	228 ± 26	254 ± 14	223 ± 22	
**Glu**	1275 ± 362	1577 ± 337	1915 ± 269	1427 ± 446	
**Gly**	734 ± 61	1103 ± 152	920 ± 40	806 ± 59	*,#
**His**	294 ± 36	419 ± 54	353 ± 33	323 ± 24	
**Ile**	53.9 ± 7.8	79.6 ± 13.2	67.5 ± 4.0	59.1 ± 3.8	
**Leu**	101 ± 9	152 ± 25	126 ± 5	108 ± 5	
**Lys**	145 ± 12	182 ± 30	152 ± 14	146 ± 11	
**Met**	15.8 ± 1.8	24.5 ± 3.9	15.6 ± 1.7	17.4 ± 1.8	
**Phe**	45.9 ± 2.3	66.6 ± 9.2	52.2 ± 1.8	51.7 ± 3.8	
**Pro**	72.6 ± 7.2	103.0 ± 22.4	80.7 ± 5.7	81.8 ± 7.4	
**Ser**	68.2 ± 8.9	125.7 ± 20.5#	112.6 ± 13.7*	77.8 ± 10.2#	
**Thr**	91.6 ± 13.3	154.1 ± 22.8#	56.2 ± 6.2	98.7 ± 11.6	*,#
**Tyr**	49.4 ± 5.7	70.1 ± 6.7#	56.6 ± 5.8	55.3 ± 9.3	
**Val**	125 ± 16	168 ± 28	152 ± 8	130 ± 15	

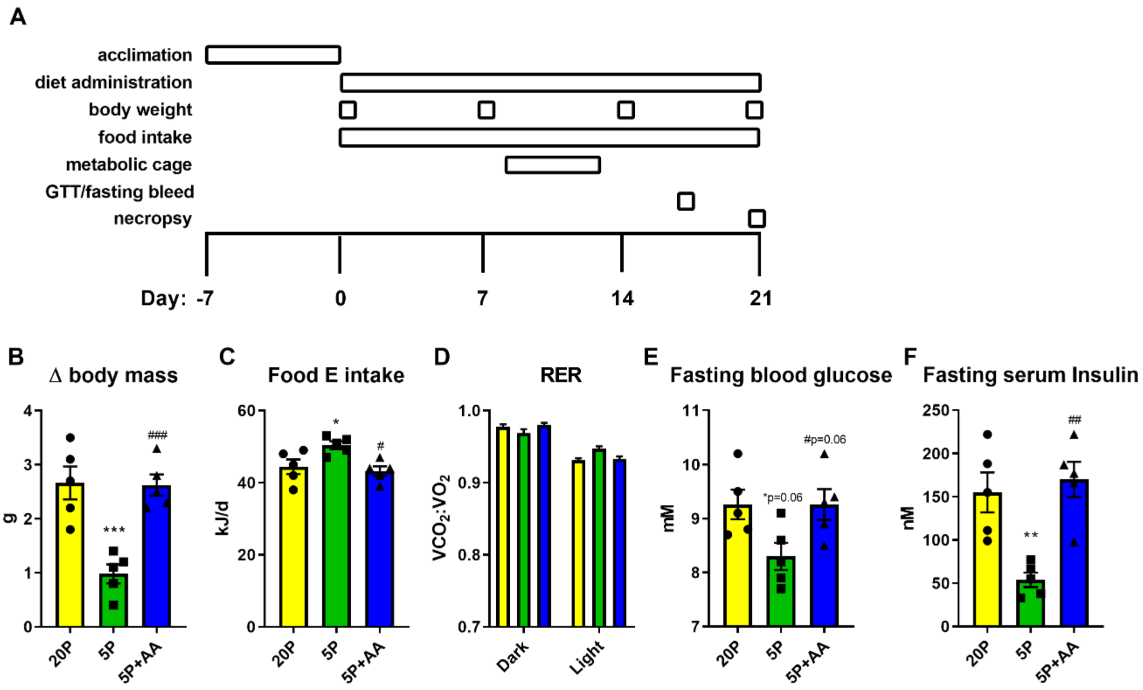

Supplementary Figure 1. Dietary amino acids are required for the systemic metabolic effects of dietary protein dilution. Related to Figure 1.

A: Protocol for the diet intervention studies.

B:: The change (Δ) in body mass of mice in response to a 3wk treatment with diets containing 20% energy from protein (20P), 5% energy from protein (5P), and 5% energy from protein and 15% energy from amino acids to match that of 20P. Data are mean and SEM; n=5 individual mice per group. Data were analysed by one-way ANOVA with Holm-Sidak post-hoc tests. Different than 20P: * P <0.05, **P<0.01, ***P<0.001. Different than 5P: $^{\#}$P<0.05, $^{\#\#}$P<0.01, $^{\#\#\#}$P<0.001.

C: The rate of food energy (E) intake during the metabolic cage housing of mice as in B.

D: The respiratory exchange ratio (VCO_2:VO_2) of mice as in B.

E: Fasting blood glucose of mice as in B.

F: Fasting serum insulin of mice as in B.

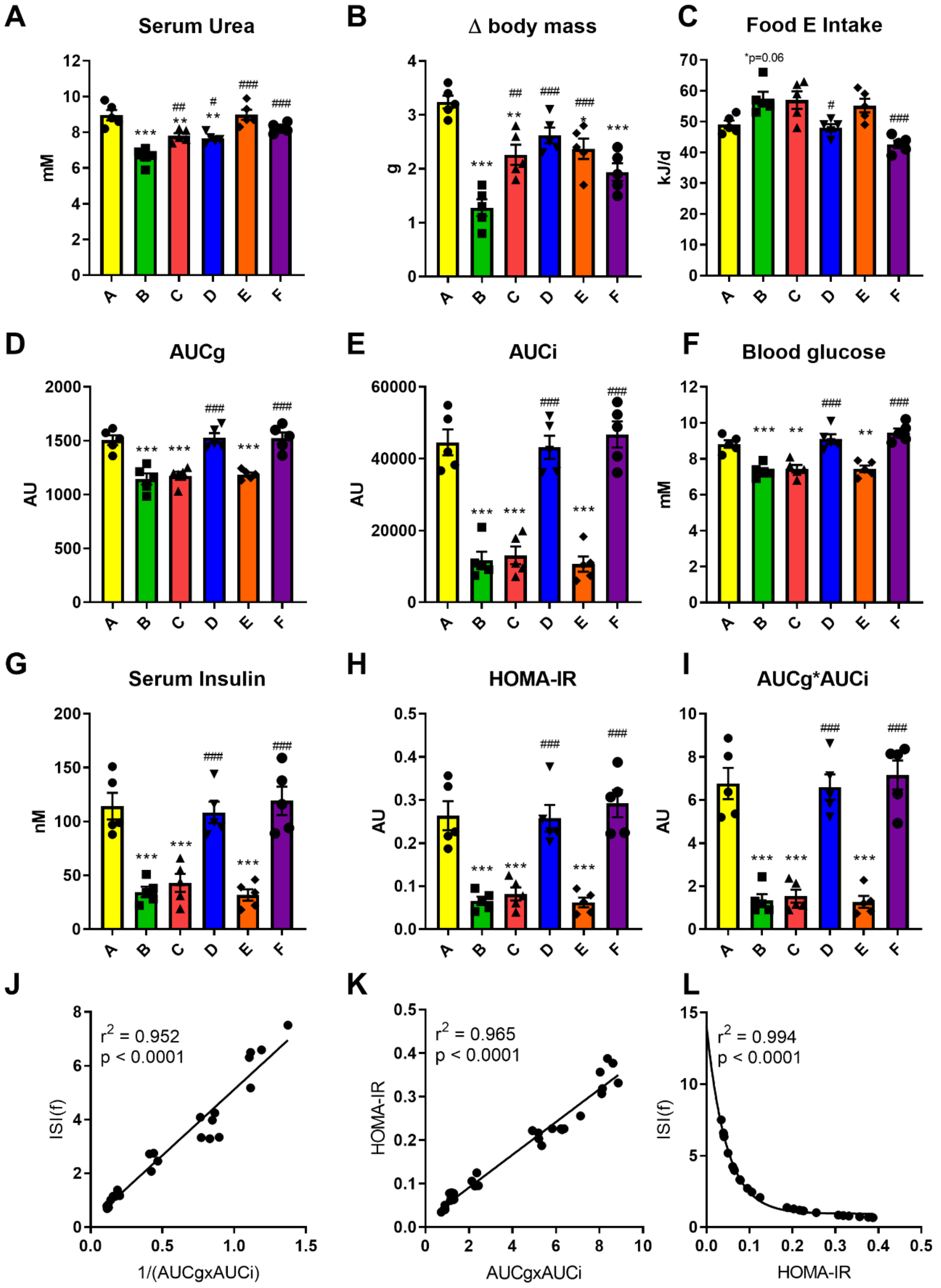

Supplementary Figure 2. Dietary essential amino acids, independent from non-essential amino acid or carbohydrate supply, dictate the systemic metabolic response to dietary protein dilution. Related to Figure 2.

A: Serum urea of mice in response to a 3wk treatment with diets as per protocol of SF1A containing nutrient energy sources as in Figure 2C. Data (N=5 individual mice per group) are mean and SEM. Data were analysed by one-way ANOVA with Holm-Sidak post-hoc tests. Different than diet A: * P <0.05, **P<0.01, ***P<0.001. Different than diet B: $^{\#}$P<0.05, $^{\#\#}$P<0.01, $^{\#\#\#}$P<0.001.

B: The change (Δ) in body mass of mice over the 3wk intervention as in A.

C: The rate of food energy (E) intake per day during the metabolic cage housing of mice as in A.

D: The glucose area under the curve (AUCg) during an intraperitoneal glucose tolerance test glucose tolerance test of mice as in A.

E: The insulin area under the curve (AUCi) during an intraperitoneal glucose tolerance test glucose tolerance test of mice as in A.

F: Fasting blood glucose of mice as in A.

G: Fasting serum insulin of mice as in A.

H: Homeostatic model assessment of insulin resistance (HOMA-IR) of mice as in A.

I: The product of the glucose and insulin areas under the curve (AUCg*AUCi) during an intraperitoneal glucose tolerance test of mice as in A.

J: A scatter plot of the fasting insulin sensitivity index (ISI(f))and inverse of the AUCg*AUCi during the intraperitoneal glucose tolerance test of mice as in A. Shown are individual data points of mice. Insert shows r^2 and p values from linear regression analysis.

K: A scatter plot of the HOMA-IR and AUCg*AUCi during the intraperitoneal glucose tolerance test of mice as in A. Shown are individual data points of mice. Insert shows r^2 and p values from linear regression analysis.

L: A scatter plot of the ISI(f) and HOMA-IR during the intraperitoneal glucose tolerance test of mice as in A. Shown are individual data points of mice. Insert shows r^2 and p values from non-linear regression analysis.

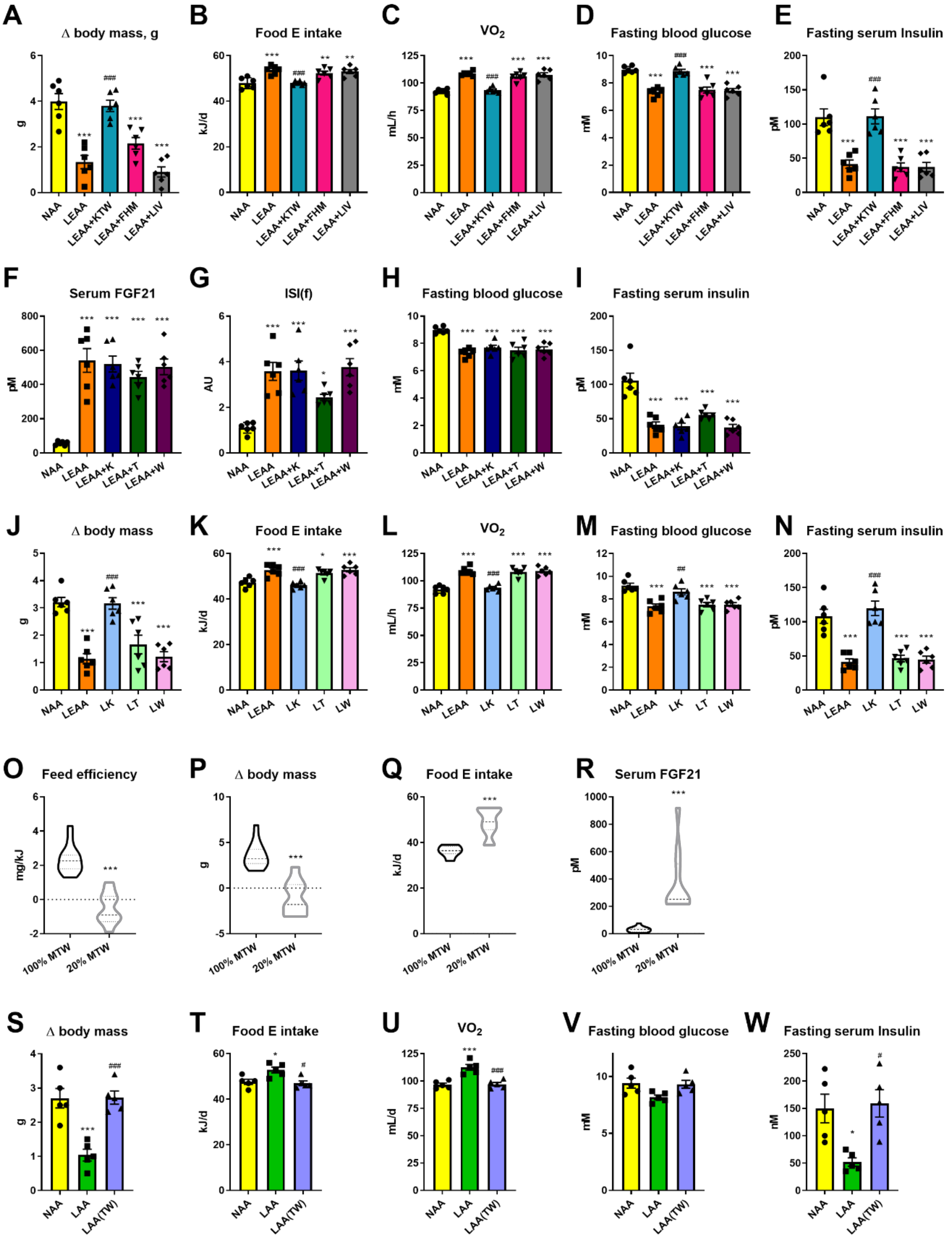

Supplementary Figure 3. Certain essential amino acids including Threonine and Tryptophan are sufficient and necessary for the systemic metabolic effects of dietary protein dilution. Related to Figure 3.

A: The change (Δ) in body mass of mice in response to a 3wk treatment with diets containing 18% from amino acids (normal amino acid; NAA as of diet A in Figure 2C), 4.5% essential AA (LEAA; as of diet E in Figure 2C), and LEAA supplemented with either lysine, threonine, and tryptophan (LEAA+KTW), phenylalanine, histidine, and methionine (LEAA+FHM), or isoleucine, leucine, and valine (LEAA+ILV), all with other AA equally adjusted to give 18%AA in total. Data are mean and SEM (n=6 individual mice per group). Data were analysed by one-way ANOVA with Holm-Sidak post-hoc tests. Different than diet NAA: * P <0.05, **P<0.01, ***P<0.001. Different than diet LEAA: $^{\#}$P<0.05, $^{\#\#}$P<0.01, $^{\#\#\#}$P<0.001.

B: The rate of food energy (E) intake during the metabolic cage housing of mice as in A.

C: The rate of O_2 consumption (VO_2) of mice as in A.

D: Fasting blood glucose of mice as in A.

E: Fasting serum insulin of mice as in A.

F: Blood glucose during fasting of mice in response to a 3wk treatment with diets containing 18% from amino acids (normal amino acid; NAA as of diet A in Figure 2C), 4.5% essential AA (LEAA; as of diet E in Figure 2C), and LEAA supplemented with either lysine (LEAA+K), threonine (LEAA+T), and tryptophan (LEAA+W), all with other AA equally adjusted to give 18%AA in total. Data are mean and SEM; n=6 individual mice per group. Data were analysed by one-way ANOVA with Holm-Sidak post-hoc tests. Different than diet NAA: * P <0.05, **P<0.01, ***P<0.001. Different than diet LEAA: $^{\#}$P<0.05, $^{\#\#}$P<0.01, $^{\#\#\#}$P<0.001.

G: Insulin sensitivity index during fasting (ISI(f)) of mice as in F.

H: Fasting blood glucose of mice as in F.

I: Fasting serum insulin of mice as in F.

J: The change (Δ) in body mass of mice in response to a 3wk treatment with diets containing 18% from amino acids (AA; NAA as of diet A in Figure 2C), 4.5% essential AA (LEAA; as of diet E in Figure 2C), and diet singly with restricted amounts of lysine (LK), threonine (LT), and tryptophan (LW), all with other AA equally adjusted to give 18%AA in total. Data are mean and SEM (n=6 individual mice per group). Data were analysed by one-way ANOVA with Holm-Sidak post-hoc tests. Different than NAA: * P <0.05, **P<0.01, ***P<0.001. Different than LEAA: $^{\#}$P<0.05, $^{\#\#}$P<0.01, $^{\#\#\#}$P<0.001.

K: The rate of food energy (E) intake during the metabolic cage housing of mice as in J.

L: The rate of O_2 consumption (VO_2) of mice as in J.

M: Fasting blood glucose of mice as in J.

N: Fasting serum insulin of mice as in J.

O: Feed efficiency of mice in response to a 6wk treatment with diets containing normal amount of methionine, threonine and tryptophan (100% MTW; n=12 individual mice) or specifically reduced levels of these amino acids (20% MTW; n=11 individual mice). Data are shown as piano plots with median (thick dashed line) and quartiles (thin dashed lines) shown. Data were analysed by Mann-Whitney rank test. Different than 100% MTW: * P <0.05, **P<0.01, ***P<0.001.

P: The change (Δ) in body mass of mice as in O.

Q: Food energy (E) intake of mice as in O.

R: Serum FGF21 of mice as in O.

S: The change of body weight of mice in response to a 3wk treatment with diets containing 18% from amino acids (normal amino acid; NAA as of diet A in Figure 2C), 4.5% AA (LAA; as of diet B in Figure 2C), and LAA supplemented with threonine and tryptophan while keeping

total AA at 4.5% (LAA(TW)). Data are mean and SEM (n=5 individual mice per group). Data were analysed by one-way ANOVA with Holm-Sidak post-hoc tests. Different than NAA: * P <0.05, **P<0.01, ***P<0.001. Different than LAA: $^{\#}$P<0.05, $^{\#\#}$P<0.01, $^{\#\#\#}$P<0.001.

T: The rate of food energy (E) intake during the metabolic cage housing of mice as in S.

U: The rate of O_2 consumption (VO_2) of mice as in S.

V: Fasting blood glucose of mice as in S.

W: Fasting serum insulin of mice as in S.

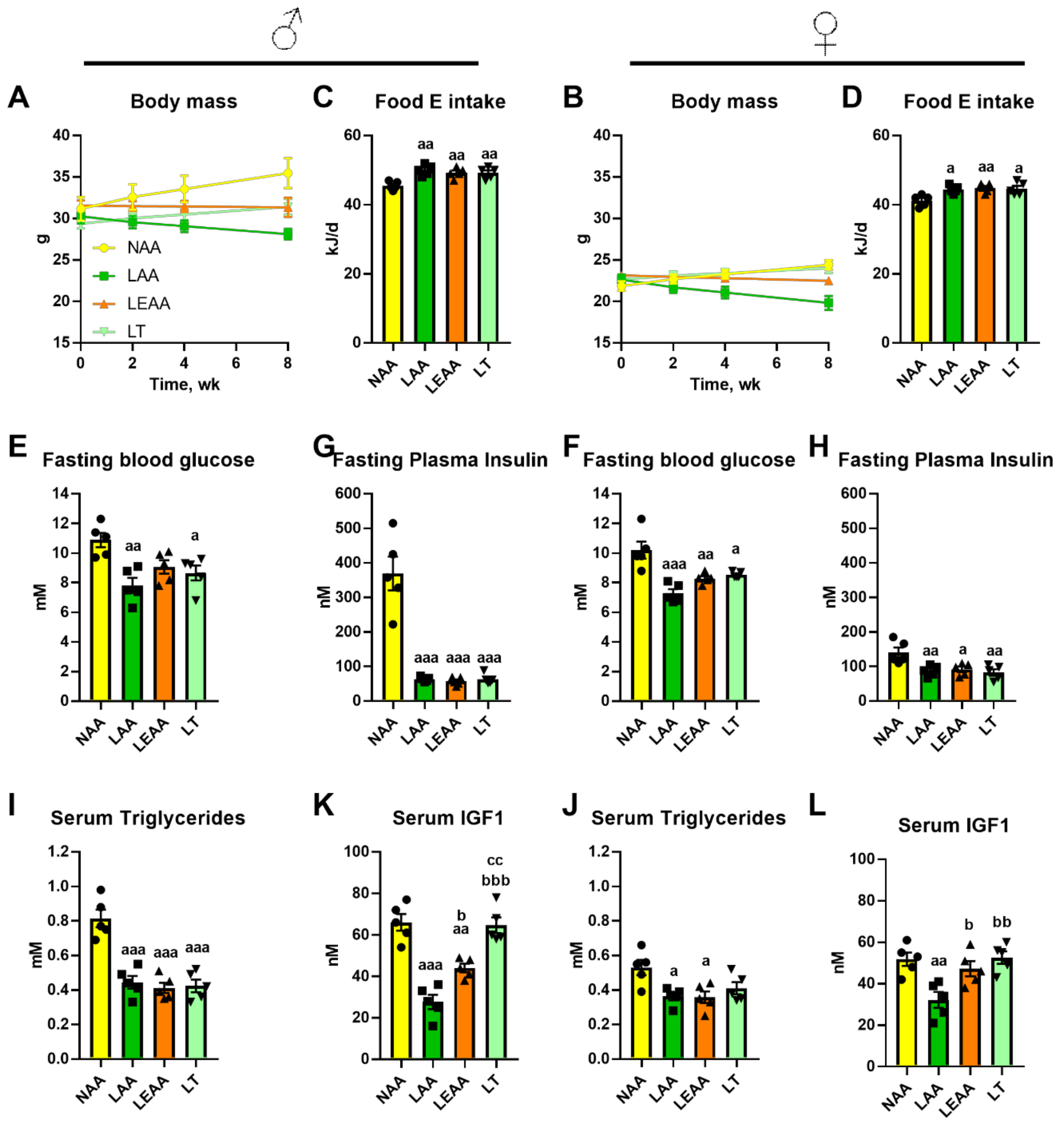

Supplementary Figure 4. The systemic metabolic response to dietary AA restriction is conserved in mature male and female mice. Related to Figure 4.

A: Body mass of 6mo old male (shown left) mice in response to an 8wk treatment with diets containing 18% from amino acids (normal amino acid; NAA as of diet A in Figure 2C), 4.5% AA (LAA; as of diet B in Figure 2C), 4.5% essential AA (LEAA; as of diet E in Figure 2C), and a diet low in Threonine but with matching total AA to NAA (as of diet LT in Figure 3E). Data are mean and SEM (n=5 individual mice per group). Data were analysed by one-way

ANOVA with Holm-Sidak post-hoc tests. Different than NAA: ^{a} P <0.05, ^{aa}P<0.01, ^{aaa}P<0.001. Different than LAA: ^{b}P<0.05, ^{bb}P<0.01, ^{bbb}P<0.001. Different than LEAA: ^{c}P<0.05, ^{cc}P<0.01, ^{ccc}P<0.001.

B: Body mass of 6mo old female (shown right) mice treated as in A.

C: The rate of food energy (E) intake of mice as in A.

D: The rate of food energy (E) intake of mice as in B.

E: Fasting blood glucose of mice as in A.

F: Fasting blood glucose of mice as in B.

G: Fasting plasma insulin levels of mice as in A.

H: Fasting plasma insulin levels of mice as in B.

I: Serum triglyceride levels of mice as in A.

J: Serum triglyceride levels of mice as in B.

K: Serum insulin-like growth factor 1 (IGF1) levels of mice as in A.

L: Serum IGF1 levels of mice as in B.

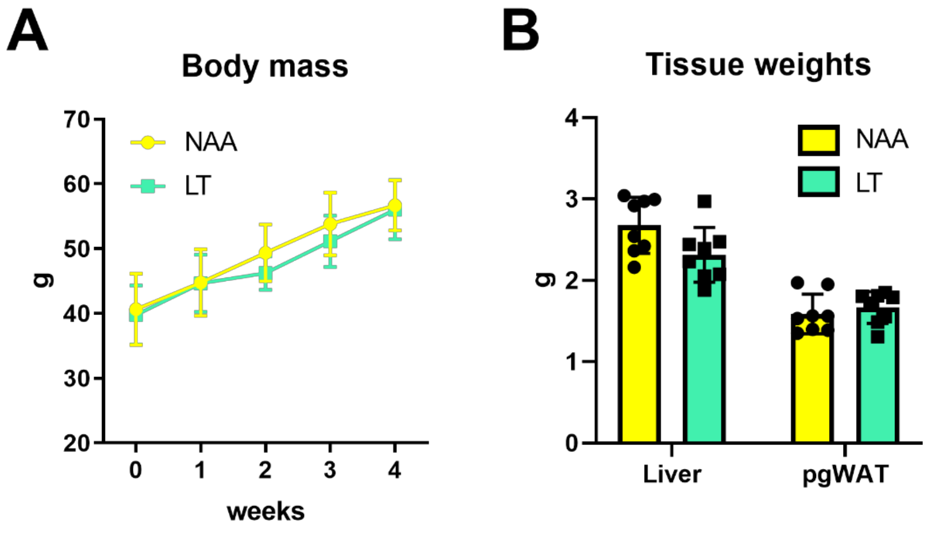

Supplementary Figure 5. Threonine restriction is a common feature of other models of systemic AA restriction and retards obesity-induced metabolic dysfunction in mice. Related to Figure 5.

A: Body mass during a 4 wk treatment of New Zealand Obese mice fed diets containing 18% from amino acids (normal amino acid; NAA) or a diet low in Threonine but with matching total AA to NAA (LT). Data are mean and SEM (N=8 individual mice per group). Data were analysed by one-way repeated measures ANOVA. Different than NAA: * P <0.05, **P<0.01, ***P<0.001.

B: Tissue mass' of mice at the end of treatment as in A. Data were analysed by students t-tests. Different than NAA: * P <0.05, **P<0.01, ***P<0.001.

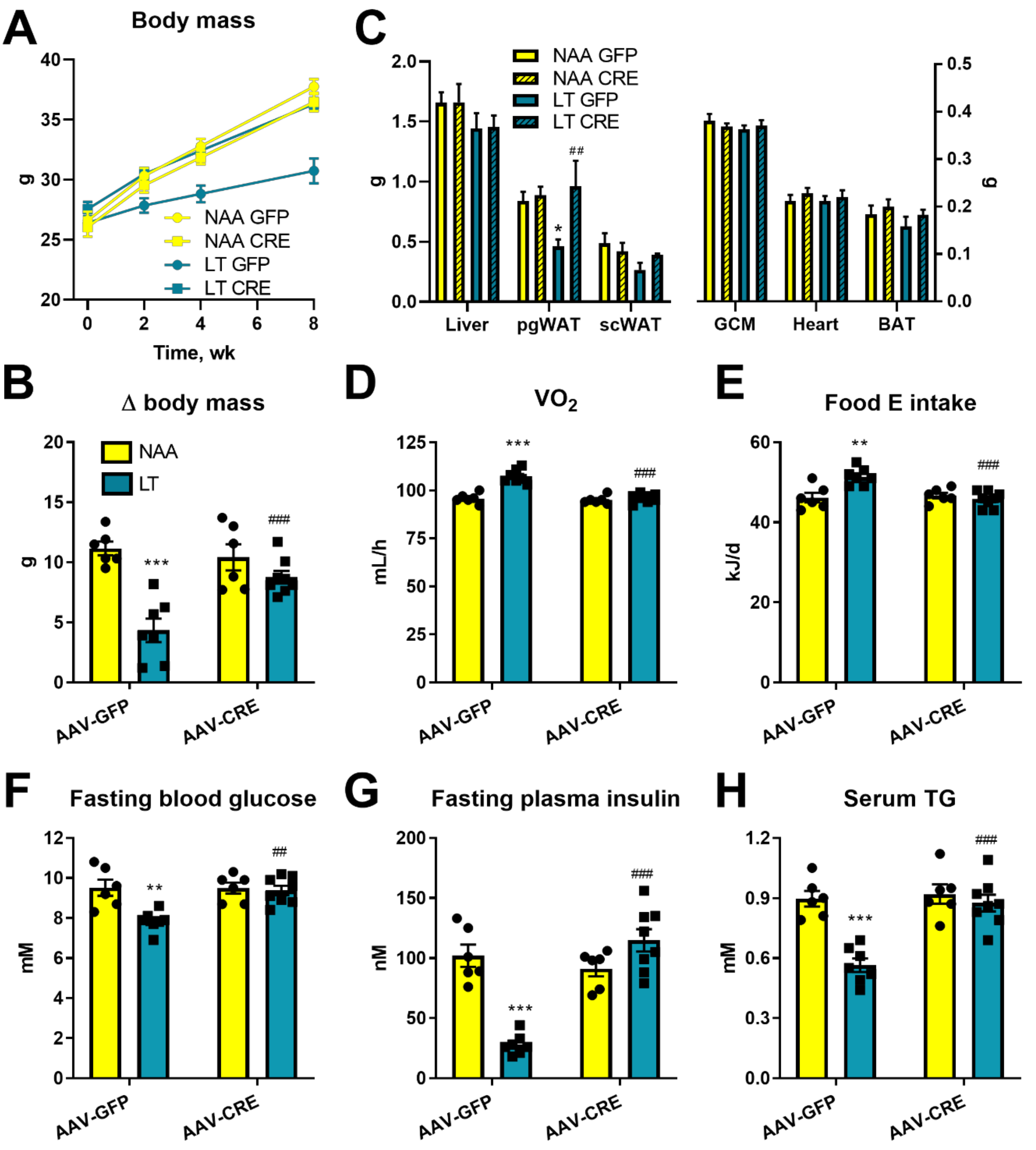

Supplementary Figure 6. Liver-derived fibroblast growth factor 21 is necessary for the systemic metabolic remodelling with dietary threonine restriction. Related to Figure 6.

A: Body mass of Fgf21fl/fl mice during an 8wk treatment with diets containing 18% from amino acids (AA; NAA) or low threonine with other AA equally adjusted to give 18%AA in total (LT); with pre-treatment with adeno-associated viruses to express Cre-recombinase (AAV-CRE) or green fluorescent protein (AAV-GFP) in an hepatocyte-selective manner. Data

are mean and SEM (n=6 NAA x AAV-GFP; n=7 LAA AAV-GFP; n=6 LT x AAV-CRE; n=8 LT x AAV-CRE).

B: The change of body mass of mice over the 8wk treatment period of mice as in A. Data were analysed by two-way ANOVA with Holm-Sidak post-hoc tests. Different than NAA: * $P < 0.05$, ** $P < 0.01$, *** $P < 0.001$. Different than AAV-GFP: $^{\#}P < 0.05$, $^{\#\#}P < 0.01$, $^{\#\#\#}P < 0.001$.

C: Tissue mass' of mice at the end of treatment as in A.

D: The rate of O_2 consumption (VO_2) of mice as in A.

E: The rate of food energy (E) intake of mice as in A.

F: Fasting blood glucose of mice as in A.

G: Fasting plasma insulin levels of mice as in A.

H: Serum triglyceride levels of mice as in A.

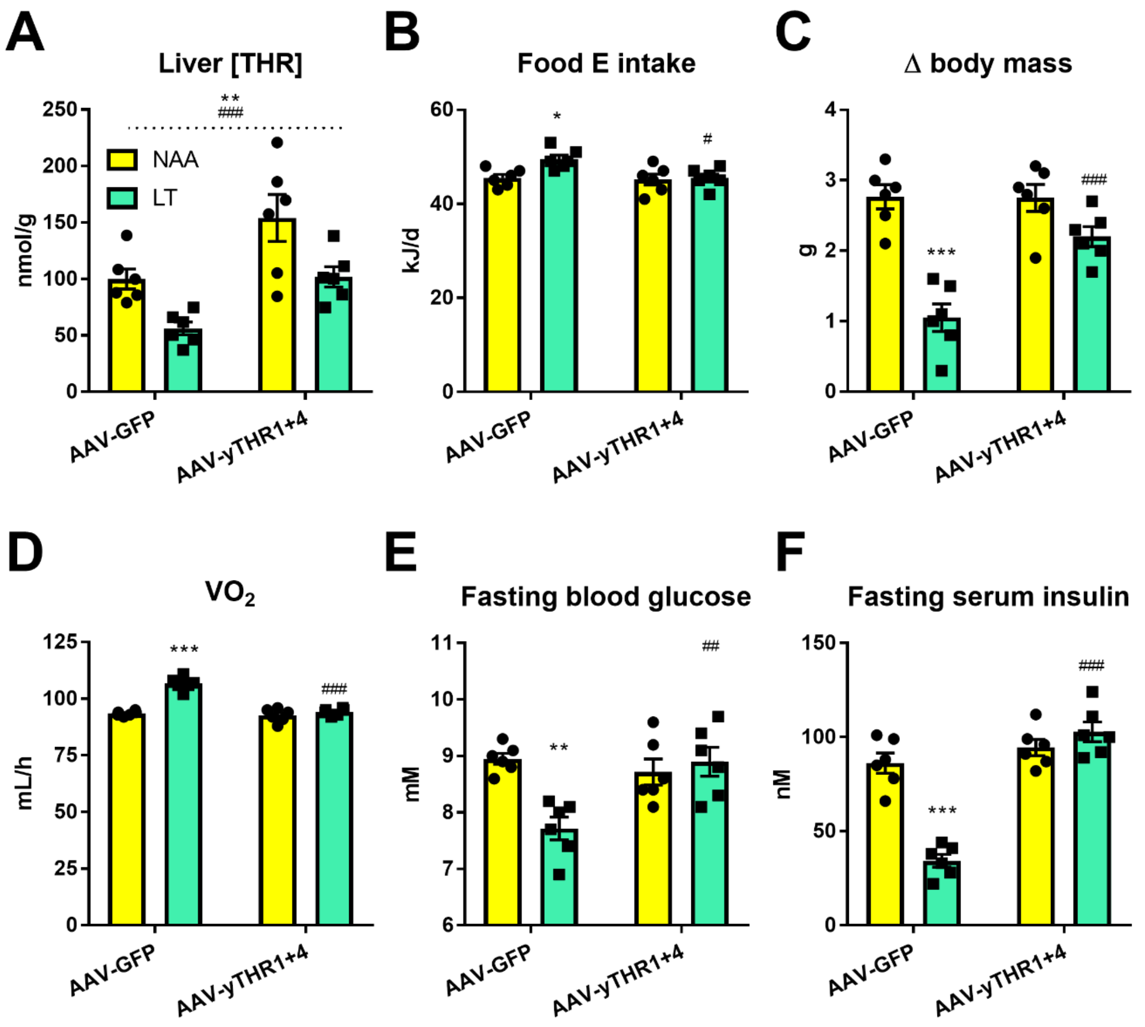

Supplementary Figure 7. Enforced hepatic threonine biosynthetic capacity reverses the systemic metabolic effects to dietary threonine restriction. Related to Figure 7.

A: Liver threonine concentration as revealed by quantitative metabolomics of mice in response to a 3wk treatment with diets containing 18% from amino acids (normal amino acid; NAA; yellow bars) and a diet with restricted amounts of threonine (LT; green bars), following prior treatments with adeno-associated viruses to transduce the liver to express yeast threonine biosynthetic enzymes (AAV-yTHR1+THR4) or a negative control (AAV-GFP). Data are mean and SEM (N= 6 individual mice per group). Data were analysed by two-way ANOVA with Holm-Sidak post-hoc tests. Different than diet NAA: * P <0.05, **P<0.01, ***P<0.001. Different than AAV-GFP: $^{\#}$P<0.05, $^{\#\#}$P<0.01, $^{\#\#\#}$P<0.001.

.

B: The rate of food energy (E) intake during the metabolic cage housing of mice as in A.

C: The change (Δ) in body mass of as in A.

D: The rate of O_2 consumption (VO_2) of mice as in A.

E: Fasting blood glucose of mice as in A.

F: Fasting serum insulin of mice as in A.
