# Supplementary material for: Restriction of essential amino acids dictates the systemic metabolic response to dietary protein dilution
Source: Nat Commun. 2020 Jun 9;11:2894. doi: 10.1038/s41467-020-16568-z (PMC7283339; doi:10.1038/s41467-020-16568-z)
Supplement: Supplementary file 3 — Description of Additional Supplementary Files [file 41467_2020_16568_MOESM3_ESM.docx]

Description of Additional Supplementary Files

**Supplementary Dataset 1:** Supplementary Metabolomics data. Annotated metabolomics data pertaining to Figure 2A-B and Suppl. Figure 7A.

**Supplementary Dataset 2:** Supplementary Proteomics data. Annotated proteomics data pertaining to Suppl. Table 3.
