# Supplementary material for: Restriction of essential amino acids dictates the systemic metabolic response to dietary protein dilution
Source: Nat Commun. 2020 Jun 9;11:2894. doi: 10.1038/s41467-020-16568-z (PMC7283339; doi:10.1038/s41467-020-16568-z)
Supplement: Supplementary file 6 — Reporting Summary [file 41467_2020_16568_MOESM6_ESM.pdf]

natureresearch

Corresponding author(s): Adam J. Rose

Last updated by author(s): 30/3/20

Reporting Summary

Nature Research wishes to improve the reproducibility of the work that we publish. This form provides structure for consistency and transparency in reporting. For further information on Nature Research policies, see Authors & Referees and the Editorial Policy Checklist .

Statistics

For all statistical analyses, confirm that the following items are present in the figure legend, table legend, main text, or Methods section.

n/a	Confirmed	
☐	☒	The exact sample size (*n*) for each experimental group/condition, given as a discrete number and unit of measurement
☐	☒	A statement on whether measurements were taken from distinct samples or whether the same sample was measured repeatedly
☐	☒	The statistical test(s) used AND whether they are one- or two-sided<br>*Only common tests should be described solely by name; describe more complex techniques in the Methods section.*
☒	☐	A description of all covariates tested
☐	☒	A description of any assumptions or corrections, such as tests of normality and adjustment for multiple comparisons
☐	☒	A full description of the statistical parameters including central tendency (e.g. means) or other basic estimates (e.g. regression coefficient) AND variation (e.g. standard deviation) or associated estimates of uncertainty (e.g. confidence intervals)
☒	☐	For null hypothesis testing, the test statistic (e.g. *F*, *t*, *r*) with confidence intervals, effect sizes, degrees of freedom and *P* value noted<br>*Give P values as exact values whenever suitable.*
☒	☐	For Bayesian analysis, information on the choice of priors and Markov chain Monte Carlo settings
☒	☐	For hierarchical and complex designs, identification of the appropriate level for tests and full reporting of outcomes
☒	☐	Estimates of effect sizes (e.g. Cohen's *d*, Pearson's *r*), indicating how they were calculated

Our web collection on statistics for biologists contains articles on many of the points above.

Software and code

Policy information about availability of computer code

Data collection	For metabolomics: IDEOM. For proteomics: Byonic v3.0.0
Data analysis	GraphPad Prism v.7.01 and SigmaPlot v.14

For manuscripts utilizing custom algorithms or software that are central to the research but not yet described in published literature, software must be made available to editors/reviewers. We strongly encourage code deposition in a community repository (e.g. GitHub). See the Nature Research guidelines for submitting code & software for further information.

Data

Policy information about availability of data

All manuscripts must include a data availability statement. This statement should provide the following information, where applicable:

- Accession codes, unique identifiers, or web links for publicly available datasets
- A list of figures that have associated raw data
- A description of any restrictions on data availability

The data that support the findings of this study are available from the corresponding author upon reasonable request.

Field-specific reporting

Please select the one below that is the best fit for your research. If you are not sure, read the appropriate sections before making your selection.

☒ Life sciences ☐ Behavioural & social sciences ☐ Ecological, evolutionary & environmental sciences

For a reference copy of the document with all sections, see nature.com/documents/nr-reporting-summary-flat.pdf

Life sciences study design

All studies must disclose on these points even when the disclosure is negative.

Sample size	Sample size was chosen based upon effect sizes outlined in our previous publication (Maida et al. JCI, 2016)
Data exclusions	No data were excluded from any analyses
Replication	Results in Figure 1, Fig 3I-L and the effects of low Thr diet were reproducible between 2 different facilities/institutes in 2 different countries/ continents using different mouse sources and 2 different metabolic cage systems.
Randomization	After acclimation, mice in cages were allocated to diet groups by counterbalancing body weights to achieve an equivalent starting body weight
Blinding	Where possible, sample analyses was blinded (metabolomics and proteomics), serum FGF21 and insulin as a distinct lab member analysed these variables unaware of the grouping variables

Reporting for specific materials, systems and methods

We require information from authors about some types of materials, experimental systems and methods used in many studies. Here, indicate whether each material, system or method listed is relevant to your study. If you are not sure if a list item applies to your research, read the appropriate section before selecting a response.

Materials & experimental systems

n/a	Involved in the study	
☒	☐	Antibodies
☒	☐	Eukaryotic cell lines
☒	☐	Palaeontology
☐	☒	Animals and other organisms
☒	☐	Human research participants
☒	☐	Clinical data

Methods

n/a	Involved in the study	
☒	☐	ChIP-seq
☒	☐	Flow cytometry
☒	☐	MRI-based neuroimaging

Animals and other organisms

Policy information about studies involving animals; ARRIVE guidelines recommended for reporting animal research

Laboratory animals	This information is fully disclosed within the methods section
Wild animals	This study did not involve wild animals
Field-collected samples	This study did not involve field collected samples
Ethics oversight	Animal experiments were conducted according to regional, national, and continental ethical guidelines and approved by local regulatory authorities (Regierungspräsidium Karlsruhe, DEU; Monash University Animal Ethics Committee, AUS; and University of Sydney Animal Ethics Committee, AUS) and conformed to ARRIVE guidelines.

Note that full information on the approval of the study protocol must also be provided in the manuscript.
